# Transcutaneous bilirubin nomograms in African neonates

**DOI:** 10.1371/journal.pone.0172058

**Published:** 2017-02-13

**Authors:** Bolajoko O. Olusanya, Cecilia A. Mabogunje, Donald O. Imosemi, Abieyuwa A. Emokpae

**Affiliations:** 1 Center for Healthy Start Initiative, Ikoyi, Lagos, Nigeria; 2 Massey Street Children’s Hospital, Lagos, Nigeria; 3 Lagos Island Maternity Hospital, Lagos, Nigeria; University College London, UNITED KINGDOM

## Abstract

**Background:**

The use of transcutaneous bilirubin (TcB) as a screening tool, based on relevant population-specific nomogram, or proxy for total serum bilirubin (TSB) levels in assessing the risk of subsequent hyperbilirubinemia is supported by several clinical guidelines on the management of neonatal hyperbilirubinemia. However, while TcB has been found to significantly over-estimate TSB in neonates of African-American ancestry, with variations across TcB devices, no nomogram has been specifically reported for this racial group. This study therefore set out to develop TcB nomograms for healthy late pre-term and term **black Af**rican neonates derived from two widely used bilirubinometers.

**Methods:**

A retrospective analysis of 12,377 TcB measurements obtained from 6,373 neonates in the first postnatal week, over a period of 48 months using Bilichek and JM-103 bilirubinometers. TcB percentiles were computed from hour-specific TcB values and nomograms developed for each of the screening devices. Predictive ability of the 75^th^ and 95th percentiles to detect significant hyperbilirubinemia was evaluated between 24–96 hours of age. The 95^th^ percentile curve was compared with those from other populations.

**Results:**

The velocity of TcB rise at 75^th^ and 95^th^ percentiles was generally higher with JM-103 than Bilichek. Both percentiles also peaked at higher TcB levels with JM-103. The 95^th^ percentile for both instruments showed a downward trend as from approximately 114 hours. Both instruments had high negative predictive values across the selected time-epochs and lower discriminatory ability than reported in non-black populations.

**Conclusions:**

The predictive utility of TcB as a potential screening tool varies across devices in black African neonates with or without risk of significant hyperbilirubinemia, and lower than levels reported in non-black populations. Equipment-specific nomograms should be considered for TcB monitoring in this racial population where TSB is not routinely available.

## Introduction

Transcutaneous bilirubin (TcB) has been widely recommended as a valuable pre-discharge screening technique for the timely identification of infants at risk of severe hyperbilirubinemia or bilirubin encephalopathy [1–3]. In poorly-resourced settings with limited or no access to laboratory facilities for plasma/serum bilirubin determination, TcB screening offers an objective risk assessment measure over visual assessment for clinical decisions for phototherapy and/or exchange transfusion [3,4]. Genetic, biological and epidemiological differences in the natural history of neonatal hyperbilirubinemia across populations have led to the emergence of population-specific nomograms for bilirubin risk assessment and monitoring in several countries [5–8]. The need to use a TcB nomogram derived from a cohort that mostly reflect the population of neonates under evaluation has also been demonstrated [8]. Although, African neonates have been frequently associated with a disproportionate risk of severe hyperbilirubinemia and kernicterus [9,10], to our knowledge, relevant TcB nomograms for this racial group have not been reported, even in developed multi-racial countries [5]. Moreover, several studies have shown that available TcB instruments tend to over-estimate total serum bilirubin (TSB) in neonates of African ancestry compared to Caucasians [11–13]. In a recent study, we also demonstrated significant disparity in the magnitude of TSB over-estimation between two most widely reported TcB devices [14]. This study therefore, set out to develop TcB nomograms based on these TcB instruments in a population of black African neonates.

## Methods

This observational study was conducted at Island Maternity Hospital (IMH) in Lagos, Nigeria, a 180-bed State run specialist maternity hospital. The hospital serves as a referral center for over 300 private and public hospitals in the Lagos metropolis and its environs.

All healthy late pre-term and full-term neonates (gestational age ≥35weeks or birthweight ≥2.2kg) delivered over a period of 48 months (December 2011 and November 2015) in the well-baby nursery were eligible for enrolment. Infants with congenital anomalies were excluded. Gestational age (in completed weeks) was based on maternal history of last menstrual period confirmed with ultrasound scan (Sonoline SI-450, Siemens, Munich) as documented in the hospital records. Eligible infants were screened between 8–10 am daily (Monday to Saturday) for jaundice using the Bilichek transcutaneous bilirubinometer (Philips Healthcare North America, Monroeville, PA) or JM-103^®^ transcutaneous bilirubinometer (Draeger Medical Telford, PA) as previously reported [14]. The Bilichek was used from 1 December 2011 to 26 November 2012 and the JM-103 from 27 November 2012 to 30 November 2015. Two units of each device were available from onset to provide backup for maintenance purposes. The Bilichek^™^ technology uses multi-wavelength (400–760 nm) in which the reflected light is analyzed over a spectrum of 221 individual narrow wavelength bands, in contrast to JM-103 which uses two-wavelengths (460 and 550 nm) with a system of dual optical pathway. Detailed algorithms and other technical features of the two devices are well documented [11,12,15].

Both instruments were used in accordance with the manufacturers’ instructions for quality control. Before each measurement, the device was prepared and calibrated daily to a standard reference by two specially trained and dedicated nurse assistants under the supervision of the corresponding/lead author. The device is then placed on the infant’s sternum while lying in the bassinet to determine the TcB level. Each device was configured to display a TcB value derived from the average of five spectral collections when the light source in the device was triggered by the tester. Each measurement lasted approximately 10 to 15 seconds, with the probe positioned in approximately the same spot for each spectral collection. After each measurement, the disposable probe in the Bilichek was replaced for the next test or switched-off, while the non-disposable probe in the JM-103 was cleaned with alcohol swab for the next baby or powered off.

Because of the high prevalence of G6PD deficiency (estimated national prevalence of 16%) in this population [16], TcB readings that exceeded 3mg/dL below the recommended postnatal age threshold for phototherapy based on the American Academy of Pediatrics (AAP) guidelines [1], were assessed for TSB within approximately one hour. While the infants were routinely returned to the hospital for the mandatory Bacillus Calmette–Guérin (BCG) immunization post-discharge, mothers were also actively encouraged to return if they observed any yellowish discoloration of the baby’s skin. No more than three TcB readings per enrolled infant taken at interval of at least 6 hours were included in the analysis. TSB measurements for eligible infants were performed on heparinized capillary blood samples drawn by heel puncture and analyzed by direct spectrophotometry using the Advanced Bilirubin Stat-Analyzer (Model BR2) (Advanced Instruments Inc, Norwood, MA).

Other variables of interest were gender, postnatal age, maternal ethnicity, infant skin color at first TcB reading and mode of feeding. Skin color was classified as light brown, medium brown or dark brown using a skin color guide for African newborns, as previously reported [14].

### Ethics statement

This study was conducted under the institutional ethical approval (Reference No: SHMB/728/Vol. VI, dated 23 January 2015) from Lagos State Health Service Commission, the Ethics Review Board of all hospitals owned and managed by the Lagos State Government of Nigeria. Informed consentfrom the parents were obtained in writing prior to enrolment of the infants using a duly approved consent form. All patient records were anonymized and de-identified prior to analysis.

### Statistical analysis

The characteristics of the enrolled infants for each of the two instruments were compared by descriptive analysis. Only measurements recorded within 0 to 168 hours after birth and prior to receiving phototherapy were eligible for analysis. All TcB measurements were categorized into 6hr-epochs from birth. As this was a retrospective evaluation of data from but not limited to previously reported prospective research on filtered sunlight phototherapy [17], infants with missing postnatal age or TcB readings were excluded from the analysis. The 10^th^, 25^th^, 50^th^, 75^th^ and 95^th^ percentiles for each time epoch were computed with IBM SPSS Statistics for Windows software, Version 23.0 (IBM Corporation, Armonk, NY). The output was migrated to Microsoft Excel 2016 software (Microsoft Corporation, Redmond, WA) to construct hour-specific nomograms for Bilichek and JM-103. Smoothened second-order polynomial trendlines that best fit the scatter plots of the percentile TcB values for each epoch were created [18]. The predictive ability of TcB measurements above 75^th^ and 95^th^ percentiles by either Bilichek or JM-103 to detect an infant with significant hyperbilirubinemia between 24 and 96 hours of age was assessed by sensitivity, specificity, positive predictive value (PPV), negative predictive value (NPV) and positive likelihood ratio (PLR). Significant hyperbilirubinemia was determined by the age-appropriate TSB levels for phototherapy as per AAP guidelines [1] and within the context of the high prevalence of G6PD deficiency in this population. The receiver operating curve (ROC) analysis was also performed to assess the ability of the nomograms for Bilichek and JM-103 to predict significant hyperbilirubinemia between 24 and 96 hours of age. The nomograms were compared to those for different racial/ethnic populations reported in other studies. All tests of statistical significance were two-tailed at 95% confidence interval.

## Results

Of the 6,451 infants enrolled, a total of 6,373 neonates with 12,377 TcB measurements were eligible for analysis. Some 4,057 (63.7%) of the infants had two TcB measurements while 1,947 (30.6%) had three TcB measurements. A total of 10,025 (81.0%) of the measurements were obtained from JM-103. The characteristics of the infants screened by either Bilichek or JM-103 are presented in Table 1. There was no statistically significant difference in male: female ratio, mean birthweight and gestational age of the infants screened by either TcB instrument. The vast majority of the infants were exclusively breastfed (80%), had light or medium brown skin color (93.7%), belonged to the Yoruba tribe (68.1%) and were enrolled between 24 and 96 hours of age (68.5%).

**Table 1:** Profile of infants enrolled for study.

Factors	Total	BiliChek	JM-103
	n = 6373 (%)	n = 1298 (%)	n = 5075 (%)
**No of TcB measurements**	12377	2352	10025
**Gender**			
Female	2983	587 (45.2)	2396 (47.2)
Male	3390	711 (54.8)	2679 (52.8)
**Birth weight**			
<2.5 kg	326	94 (7.3)	232 (4.6)
2.5–3.0 kg	2144	458 (35.5)	1686 (33.5)
>3.0 kg	3860	739 (57.2)	3121 (61.9)
Missing data	43	7 (0.5)	36 (0.7)
Mean (± Standard deviation)	3.22 **±** 0.50	3.18 **±** 0.52	3.22 **±** 0.49
**Gestational age**			
<35 weeks	99	27 (2.1)	72 (1.5)
35–37 weeks	891	157 (12.2)	734 (15.2)
>37 weeks	5130	1104 (85.7)	4026 (83.3)
Missing data	253	10 (0.9)	243 (4.8)
Mean (± Standard deviation)	38.40 **±** 1.59	38.17 **±** 1.52	38.46 **±** 1.61
**Postnatal age**			
0–24 hours	1607	318 (24.5)	1289 (25.4)
24.1–48 hours	2720	550 (42.4)	2170 (42.8)
48.1–72 hours	1131	202 (15.6)	929 (18.3)
72.1–96 hours	516	106 (8.2)	410 (8.1)
Above 96 hours	399	122 (9.4)	277 (5.5)
**Ethnicity**			
Hausa	309	69 (5.3)	240 (4.7)
Igbo	929	176 (13.6)	753 (14.8)
Yoruba	4340	878 (67.6)	3462 (68.2)
Others	795	175 (13.5)	620 (12.2)
**Skin color**			
Light Brown	3493	685 (52.8)	2808 (55.3)
Medium Brown	2476	494 (38.1)	1982 (39.1)
Dark Brown	404	119 (9.2)	285 (5.6)
**Feeding mode**			
Exclusive breast milk	5032	1162 (89.5)	3870 (76.3)
Breast milk with formula	741	76 (5.9)	665 (13.1)
Formula only	600	60 (4.6)	540 (10.6)

**Fig 1:**
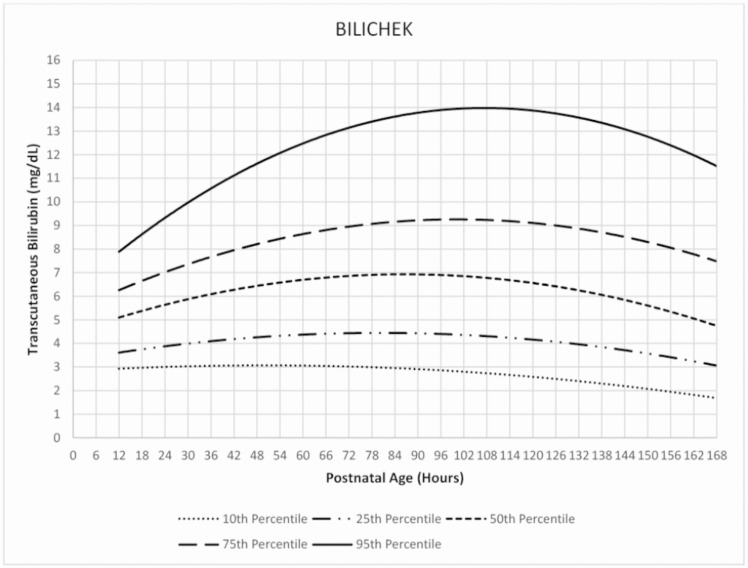
Hour-specific nomogram for transcutaneous measurements with Bilichek bilirubinometer in African neonates.

**Fig 2:**
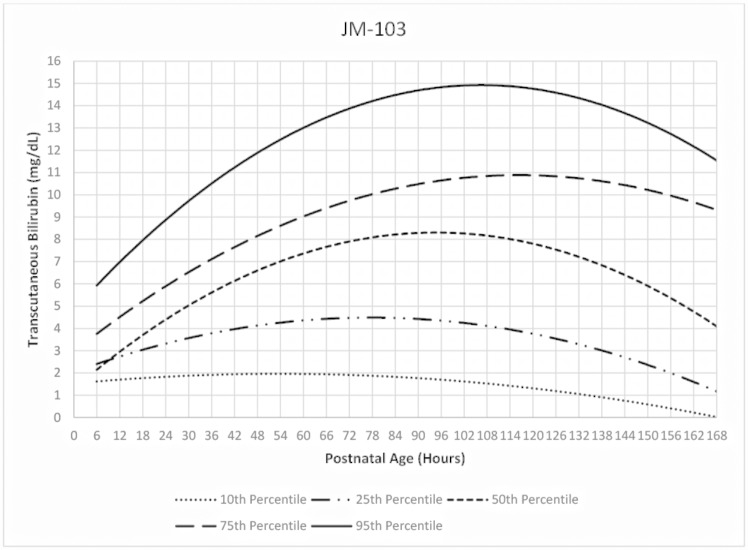
Hour-specific nomogram for transcutaneous measurements with JM-103 bilirubinometer in African neonates.

TcB: transcutaneous bilirubin; TSB: total serum bilirubin;

The TcB nomograms for infants screened in the first week of life with Bilichek or JM-103 from ages 6 to 168hours are presented in Figs 1 and 2. The rates of rise of the 50^th^, 75^th^ and 95^th^ percentiles were generally higher with the JM-103 than the Bilichek. They also peaked at higher TcB levels. The 95^th^ percentile for both instruments showed a downward trend as from approximately 114 hours of age.

The predictive ability of TcB measurements above the 75^th^ and 95^th^ percentiles between 24 and 96 hours of age based on the instrument used is summarized in Table 2. This analysis was based on 1,828 paired TcB-TSB measurements from 1,034 neonates. The highest sensitivity of 100% was recorded only at the 75^th^ percentile, by JM-103 between 48 and 96 hours of age, and by Bilichek between 72 and 96 hours. Both instruments were associated with high NPV (73% to 100%) across the selected time epochs. The PLR for the 95^th^ percentile was consistently higher than the level at the 75^th^ percentile for all time epochs. The area under the ROC for JM-103 (c-statistic: 0.759, 95% CI: 0.719–0.800) was higher than the value for Bilichek (c-statistic: 0.696, 95% CI: 0.630–0.763), but the difference was not statistically significant (p = 0.142).

**Table 2:** Ability of TcB measurements above the 75^th^ and 95^th^ percentiles of TcB nomogram to predict significant hyperbilirubinemia for designated time periods.

Time period/Percentiles	Sensitivity	Specificity	PPV	NPV	PLR
	%, (95% CI)	%, (95% CI)	%, (95% CI)	%, (95% CI)	(95% CI)
**BILICHEK Nomogram**					
**24.0–96.0 Hrs**					
Above 95th	34.9	93.4	65.9	79.7	5.3
	(24.8–46.2)	(89.3–96.3)	(50.1–79.5)	(74.4–84.4)	(3.0–9.4)
Above 75th	90.4	30.8	32.3	89.7	1.3
	(81.9–95.8)	(24.9–37.3)	(26.4–38.8)	(80.8–95.5)	(1.2–1.5)
**24.0–48.0 Hrs**					
Above 95th	37.7	95.5	76.7	79.7	8.4
Above 75th	90.2	41.7	37.7	91.5	1.6
**48.1–72.0 Hrs**					
Above 95th	25.0	89.1	50.0	73.2	2.3
Above 75th	90.0	8.7	30.0	66.7	1.0
**72.1–96.0 Hrs**					
Above 95th	50.0	88.0	25.0	95.7	4.2
Above 75th	100	4.0	7.7	100.0	1.0
**JM-103 Nomogram**					
**24.0–96.0 Hrs**					
Above 95th	76.6	81.1	29.9	97.0	4.1
	(68.8–83.2)	(78.9–83.1)	(25.3–34.9)	(95.9–97.9)	(3.5–4.7)
Above 75th	98.6	17.8	11.2	99.2	1.2
	(95.1–99.8)	(15.8–19.9)	(9.6–13.1)	(97.1–99.9)	(1.1–1.3)
**24.0–48.0 Hrs**					
Above 95th	75.3	89.1	48.7	96.3	6.9
Above 75th	97.9	30.7	16.3	99.1	1.4
**48.1–72.0 Hrs**					
Above 95th	78.8	76.2	19.4	98.0	3.3
Above 75th	100	5.1	7.1	100.0	1.1
**72.1–96.0 Hrs**					
Above 95th	80.0	65.1	13.8	97.9	2.3
Above 75th	100	2.3	6.7	100.0	1.0

PPV: Positive Predictive Value; NPV: Negative Predictive Value; PLR: Positive Likelihood Ratio; TcB: Transcutaneous bilirubin, TSB: Total serum bilirubin, CI: Confidence Interval

A comparison of the high-risk zone (95^th^ percentile) in the nomograms from different population or ethnic groups is presented in Table 3, along with the type of instrument used [5,6,19–24]. The peak TcB ranged from 12.2 mg/dL to 15.5 mg/dL occurring between 90 and 108 hours of age.

**Table 3:** Comparison of 95^th^ percentile values across selected populations.

Country	Study [Reference]	TcB Meter	TcB (mg/dL) at postnatal age				Approximate Peak TcB	
			24hrs	48hrs	72hrs	96hrs	mg/dL	Age (hrs)
**Nigeria**	Olusanya et al, 2017 (Current study)	Bilichek	9.1	11.7	13.1	13.9	14.0	102
Brazil	Draque et al, 2011 [19]	Bilichek	8.0	10.5	12.0	12.5	12.2	96
Italy	De Luca et al, 2008 [20]	Bilichek	11.8	13.0	14.8	15.0	15.0	78
Greece	Fouzas et al, 2010 [21]	Bilichek	8.2	11.3	13.7	14.9	15.0	108
**Nigeria**	Olusanya et al, 2017 (Current study)	JM-103	8.2	12.0	14.0	14.7	15.0	102
Mongolia	Akahira-Azuma et al, 2015 [6]	JM-103	8.5	12.8	15.4	15.7	n/a	n/a
China	Yu et al, 2011 [22]	JM-103	7.3	9.6	14.6	16.6	14.5	108
Israel	Bental et al, 2009 [23]	JM-103	8.0	11.0	13.0	14.0	15.5	108
USA	Engle et al, 2009 [24]	JM-103	7.6	11.0	12.4	n/a	n/a	n/a
USA	Maisels et al, 2006 [5]	JM-103	7.3	10.0	12.3	13.2	13.2	90

TcB: Transcutaneous bilirubin; n/a: not available

## Discussion

In our earlier report, we demonstrated the pattern and predictors of TSB over-estimation in exclusively black African neonates using Bilichek and JM-103, and the need for further technological improvements for both devices in this population [14]. While these improvements are being developed, the lack of TSB monitoring devices makes the use of TcB inevitable in resource-constrained settings. Within this context, the present study essentially reports TcB nomograms for black African neonates and explores possible impact on the choice of bilirubinometer used for identifying infants at risk of significant hyperbilirubinemia in this population.

Studies in multi-racial populations have suggested that neonates of African ancestry are likely to have a lower overall risk of significant hyperbilirubinemia but a higher and disproportionate burden of bilirubin encephalopathy [25]. However, the high prevalence of hemolytic disease such as G6PD deficiency, frequently exacerbated by (TA)n promoter polymorphism of the urine-diphosphate-glucuronosyltransferase 1A1 gene (UGT1A1) in this ethnic population mandates early detection and monitoring of infants with significant hyperbilirubinemia [16]. Visual estimation by cephalocaudal progression is still common among clinicians especially in resource-limited settings, but has been shown to correlate poorly with TSB levels [26], thus making TcB the most viable available alternative so far. Although, TcB devices are still not widely used in developing countries, where available, this study highlights potential trade-offs in the choice of device, consistent with findings in other populations where under-estimation is more prevalent [27,28].

The predictive ability of TcB nomograms in a multi-racial population was first explored by Bhutani et al using the Bilichek [11]. The study proposed 75^th^ percentile TcB cut-off for accurately identifying infants at risk of significant hyperbilirubinemia, based on the corresponding >95^th^ percentile levels on the hour-specific bilirubin nomogram. Maisels & Kring subsequently demonstrated the need to characterize the natural history of bilirubinemia in newborns highlighting the critical role of TcB values at the 95^th^ percentile in tracking infants at risk of significant hyperbilirubinemia in the first 96 postnatal hours in a multi-racial cohort of infants [5]. However, the predictive ability of the nomogram they developed with JM-103, was not evaluated. We therefore, chose to specifically evaluate our nomograms based on 75^th^ and 95^th^ percentiles.

Our study suggests that while the 75^th^ percentile for both instruments had high sensitivity, it was equally associated with significant levels of false-positive rates, which may be burdensome and undesirable for resource-constrained settings. The high NPV for the 75^th^ and 95^th^ percentiles also suggest that our nomograms for the two instruments can reliably estimate infants who are unlikely to develop significant hyperbilirubinemia from 24 to 96 hours of age. Romagnoli and colleagues were perhaps the only researchers that have explored the predictive ability of nomograms derived from both JM-103 and Bilichek [29]. The measurements below 75^th^ percentiles from both instruments were found to be highly predictive of infants who were unlikely to develop significant hyperbilirubinemia between 24 and 96 hours of age (NPV: 98.4 to 100%). However, this study was among a cohort of 298 European neonates on whom both devices were used within an interval of 5 minutes. In contrast, each infant in our study was exposed to either Bilichek or JM-103, and no significant demographic differences were observed between the two groups of infants. In their study among 628 Israeli neonates with 1,091 measurements using JM-103, Bental et al also found TcB at the 75^th^ percentile cut-off to be associated with high NPV [23]. In a cohort of 6,035 Chinese neonates with 36,921 TcB measurements using JM-103, the 95^th^ percentile curve had 26.9% sensitivity and 87.5% NPV in detecting infants with significant hyperbilirubinemia defined as TSB above the 95^th^ percentile in AAP guidelines [7]. The 75^th^ percentile had 78.7% sensitivity and 98.5% NPV. Another study from India where TcB was measured by Bilichek, found the 75^th^ and 95^th^ percentiles of TcB taken within the first 48 hours of life to have NPV of between 87.5% to 95.1% [30]. The added advantage of likelihood ratio over sensitivity, specificity, negative and positive predictive values as a summary clinically relevant statistical index for predicting significant hyperbilirubinemia has been previously demonstrated [31,32]. The higher PLR for the 95^th^ percentile appears to offer a better TcB track for monitoring infants at risk of significant hyperbilirubinemia among African neonates.

The 95^th^ percentile curve for Bilichek was consistently higher than the curve for Brazilian neonates [19], and consistently lower than the 95^th^ percentile curve for the Italian neonates [20]. The difference with the Brazilian study may be attributed to the sole enrolment of exclusively breastfed neonates, while the inclusion of TcB recordings from neonates who required phototherapy may have accounted for the difference with the Italian study. Except for the Chinese neonates [22], the 95^th^ percentile curve derived from JM-103 was consistently higher than those for Mongolia [6], Israel [23], and USA [5,24]. Maisels and Kring also acknowledged that the 95^th^ percentile in their study was lower than that reported in comparable studies for unknown reasons, and provided no sub-group analysis for African-Americans to aid comparison with the current study [5].

As previously reported, JM-103 is associated with a higher imprecision (mean bias) than BiliChek, and increases as TSB levels increase [14]. This would explain the rapid rates of increase in TcB at the 75^th^ and 95^th^ percentiles for JM-103. This is corroborated by the higher AUC for JM-103 than Bilichek in predicting infants with significant hyperbilirubinemia. However, the discriminatory power of both instruments are lower than levels (AUC: 0.766 to 0.971, pooled AUC: 0.819) generally reported in non-black populations [7]. The variations across studies also underscore the need for population-specific nomograms, especially because of the pattern of under- or over-estimation by each device. We are unable to recommend a particular device over the other without appropriate cost-effectiveness analysis. The trade-offs between both devices are perhaps better left to the clinical judgement of the clinicians in each setting. We did not explore the impact of skin color on nomogram, as this factor had been previously established to have no confounding effect on TcB in this racial population [14].

Notable strengths of this study include the large population of enrolled infants, the number of TcB measurements, and the wide postnatal age covered. We were able for example, to demonstrate the trajectory of bilirubin levels beyond the 4^th^ day (96 hours) of life to make it possible to assess the high proportion of infants who typically are born outside hospitals and present with significant hyperbilirubinemia from this age. However, a few limitations are worth noting. For example, despite the large sample size, the findings from this single hospital would require further validation in a multi-center study to facilitate satisfactory generalization at the population level. The predictive ability of the nomograms was limited to a proportion of infants for which paired TcB and TSB values were available, which may be subject to bias. However, for ethical reasons, we could not obtain TSB measurements for all otherwise healthy infants with no signs or apparent risk of jaundice. While it is not uncommon to use repeated TcB measurements for some infants and only one for others in developing nomograms, this approach may be subject to selection bias. Differences in methodology, site of TcB measurement (sternum versus forehead), variability in inter-laboratory TSB determination, criteria for significant hyperbilirubinemia and across racial groups may have constrained effective comparison with other studies. We did not specifically compare the performance of both units of the same TcB device at the start of project besides the daily calibration to ensure that the devices functioned within the allowable quality control limits before deployment. Lastly, our nomograms should ideally, be validated in an independent sample of neonates which was not possible. Notwithstanding, our study addresses a critical gap for the early detection of infants with or without the risk of significant hyperbilirubinemia in resource-limited African settings where routine TSB estimation is not immediately available. It should also be of interest for the care of neonates of African ancestry in more developed nations. The potential use of TcB in combination with relevant clinical risk factors to enhance its predictive utility merits future investigation in this population [33,34].

## Conclusion

This study explores the natural course of TcB levels in an exclusive cohort of black African neonates using Bilichek and JM-103 bilirubinometers. The moderate positive likelihood ratios (>5) for both instruments and the excellent negative predictive values at the 95^th^ percentile in the first 48 hours of life suggest some utility for the appropriate use of TcB to estimate TSB in resource-limited settings before hospital discharge. However, the limitations of TcB devices, and differences between nomograms derived from the devices in predicting significant hyperbilirubinemia in the first postnatal week should be recognized to facilitate effective treatment and optimal outcomes in this racial group. Additionally, the use of TcB nomograms developed from non-black population would appear inappropriate for neonates of African ancestry.

## Abbreviations

AAP: American Academy of Pediatrics
G6PD: Glucose 6-phospho-dehydrogenase
IMH: Island Maternity Hospital
PPV: Positive predictive value
NPV: Negative predictive value
PLR: Positive likelihood ratio
OR: Odds ratio
TcB: transcutaneous bilirubin
TSB: Total serum/plasma bilirubin
ROC: Receiver operating characteristic curve
AUC: Area under the receiver curve
